# Case of Popliteal Aneurism, in Which Secondary Hemorrhage Occurred, from the Ligature Being Thrown off by the Artery

**Published:** 1826-05

**Authors:** P. M. Benja

**Affiliations:** Medical Staff.


THE LONDON
### Medical and Physical Journal.

No. 5 OF VOL. LV.] MAY, 1826. [No. 327.

For many fortunate discoveries in medicine, and for the detection of numerous errors, the world is indebted to the rapid circulation of Monthly Journals; and there never existed any work, to which the Faculty, in Europe and America, were under deeper obligations, than to the Medical and Physical Journal of London, now forming a long, but an invaluable, series.—RUSH.

### ORIGINAL COMMUNICATIONS,
### SELECT OBSERVATIONS, &c.

ART. I.—*Case of Popliteal Aneurism, in which secondary Hemorrhage occurred, from the Ligature being thrown off by the Artery.* By P. M. BENJA, M.D. Medical Staff.

GIOVANNI MAUNNONI, æt. fifty, a native of Messina, has followed the employment of cook and private servant to officers and gentlemen in the Ionian Islands, for the last ten years. Last winter, while at Zante, his avocation obliged him to be exposed to a strong fire the greatest part of the day, for many weeks together. In February, 1825, he began to feel an uneasy sensation of weariness in the ham of the left leg; which, however, either diminished greatly or ceased altogether at night.

After a fortnight, he perceived for the first time a small tumor in the ham, the size of a hazel-nut, and throbbing strongly. The increase of this tumor, however, was very slow for the first two months, and did not give him much uneasiness. The patient states that a local practitioner applied the tourniquet at that period, some hours every day, for many weeks, but without any visible diminution of the tumor. The leg, however, was not œdematous, and the patient could walk without much pain or fatigue.

Seeing that his complaint could not be cured without an operation, he embarked for Italy for that purpose. The vessel put into Prevesa, where he landed. The tumor then had increased in size, and he could not walk with the same freedom as before embarkation.

When he arrived at Corfu, the leg was œdematous in a slight degree, but he could walk on crutches. Unfortunately, he was obliged to perform twenty days' quarantine, during which time I saw him occasionally in the lazaretto, suffering excruciating pains in the knee and leg: the tumor was increasing rapidly, and the leg and foot had become œdematous to a considerable extent.

Two days after he had taken pratique, he was admitted into my hospital; and then the case offered the following symptoms and appearances:—

Great emaciation: total loss of appetite, but in good spirits, and in full confidence of being saved by the operation; leg bent to nearly a right angle with the thigh, so that, when standing, there was fully a foot distance from the ground to the toes; a large irregular tumor in the ham of the left leg, measuring twenty-four inches (the knee included) in circumference, having displaced the tendons of the vasti, semi-tendinosus, and semi-membranosus muscles; the skin discoloured in the most prominent part of the tumor, its pulsation being deep and dull; leg and foot œdematous; articular arteries of the knee pulsating strongly. The patient suffers occasional, but severe, pains in the knee and leg; he is free from any fever. Temperature of the affected limb higher than that of the sound one, the former being 100°, and the latter 96°.

I showed the case to Dr. Hennen, inspector of hospitals, who encouraged me to undertake the operation; and I performed it accordingly on the morning of the 21st August, 1825, in presence of the inspector of hospitals, of Dr. Ryan of the 51st regiment, and some of the local practitioners. My friend, Mr. Roe, surgeon of the 28th, gave me his valuable assistance.

I made the incision on the upper third of the thigh, and having exposed the femoral artery for nearly an inch and a half, and carefully separated it from the accompanying vessels and nerves, I passed a double ligature under it, by means of a common aneurism needle. The ligatures were made of two threads each of common white silk; both ligatures were brought close to where the artery was still enveloped by the surrounding parts. The moment the upper ligature was drawn tight, the dull pulsation of the tumor ceased. Both ends of both ligatures were cut close to the knots, and the artery divided between them. The edges of the wound were brought together by straps of adhesive plaster, and a roller moderately tight was applied.

During the operation the patient had been faint, but more from fear than from loss of blood, having lost hardly two ounces. After having been removed to bed, he complained of a burning heat in the foot of the operated leg; said that he did not any longer feel the throbbing sensation in the tumor, which used to distress him betimes. Temperature of the leg nearly natural; bowels were opened this morning.

After two hours from the operation, the dressings became stained with arterial blood; but, it being in a very small quantity, and immediately checked by cold applications, it was

6

thought to proceed from some small subcutaneous arterial branches.

Vespere.—Complains of pain in the tumor, and of a sensation as if blood was descending from it towards the wound; the toes of the affected leg rather cold, but the foot and the leg of a natural temperature; complains of a painful throbbing in the wound. A bag of warm sand was applied to the foot. Tea and toast. to the stomach.

℞. Tinct. opii, ʒss.

Aquæ menth. pip. ℥jss. M. fiat haustus hora somni sumendus.

August 23d.—Feels occasional and severe pains in the outside of the knee; tumor feels softer, and its circumference diminished by a few lines; limb hotter than the sound one. Complains of a sensation as if *lightning* [his own expression,] was passing from the knee to the wound.

August 24th.—Dressings removed. Skin of the wound healed; a small non-pulsating tumor was observed about the middle of the wound. At eleven o'clock at night, a most alarming hemorrhage took place; and, had it not been for the immediate assistance afforded by the person left to watch him, by pressing on the artery above the wound, the patient must have inevitably bled to death. Notwithstanding prompt assistance, he lost nearly three pounds of blood, which, in the exhausted state of the patient, induced great debility.

After some trouble, and with the assistance of Mr. Roe, the artery was secured again. The small tumor, which had been observed in the morning, was formed of coagulated blood, oozed from the upper end of the divided artery. Among the clots of blood, I could not discover the ligature, the casting-off of which had given rise to the hemorrhage.

September 8th.—From this time to the 8th September, he went on improving, and the slight discharge from the wound had nearly ceased: more than two-thirds of it had healed.

The emaciation of the patient is still very great, and such that the femoral artery *is seen* pulsating from the arch of the Poupart to the place where generally the profunda is given off; but, lower down, the pulsation is neither seen nor felt. A few small discoloured spots along the skin.

The outside of the heel, on which the whole limb has rested for many weeks, is very much discoloured, and some small spots along the outer margin of the foot. Œdema of the limb undiminished; sensation of it perfect, with the exception of the dorsum pedis, where it is rather dull; great toe colder than the remainder of the limb.

Sept. 12th.—Seeing that, notwithstanding the healthy appearance of the wound, the black gangrenous spot of the heel was enlarging,—the œdema still the same,—the tumor stationary of late, although softer than before,—to remove all obstacles to the circulation of the limb, and to the free action of the lymphatics, I opened the aneurismal sac, and having broken down with my finger all the coagula of blood, I extracted some, and at each dressing forced out a great quantity of tough flesh-like fibrine.

Sept. 17th.—The sac was completely emptied of all its contents, and the œdema of the limb began to subside. The black spots on the shin are suppurating, and the edges of the gangrene in the heel are inflamed. The operation wound nearly healed.

Sept. 18th.—Symptoms of dysentery, with fever, dry tongue, and pervigilium. By the means of calomel and castor-oil, great quantities of bloody fæces were discharged. These symptoms continued very violent till the 20th, when they began to abate. The stools assumed a deep green colour, and lessened in frequency. The discharge from the sac was copious, purulent, and very offensive. No suppuration as yet round the gangrenous heel. Hot poultices and spirituous embrocations were made use of to hasten the separation of the slough.

Sept. 22d.—The operation wound has been healed these two days; but this morning, on removing the adhesive plaster, the centre of the cicatrix was exquisitely sensible, and, on separating its newly united margins, about two ounces of thin fœtid pus escaped. Suppuration from the sac copious, but healthy.

℞. Decoct. cinch. ℥iij. ter in die. Wine at his dinner.

Sept. 25th.—The bowel complaint ceased. Discharge from the sac lessening, thicker, and not so offensive as before. No suppuration in the heel.

October 2d.—Discharge from the operation wound very trifling, that from the sac still healthy. The suppuration begun round the gangrene in the heel. All the other suppurating spots along the shin and foot healed. Foot still œdematous; not so the leg. Allowed to sit up in an arm-chair two hours a-day.

Oct. 20th.—Operation wound healed. The sphacelus of the heel cast off, and healthy granulations springing up. No discharge from the sac, its sides having adhered.

Oct. 30th.—A small abscess formed on the centre of the cicatrix of the operation wound, and, after a few days, one of the ligatures was discharged, apparently unaltered. The small abscess healed next day. He has been walking on crutches for nearly three weeks.

December 1st.—Another small abscess formed in the same place of the cicatrix, which, on the 18th of the same month, discharged another of the ligatures.

He has been now out of hospital these four weeks, (January the 20th,) walking on crutches, and better in health than before his being taken ill with aneurism. When standing, he can touch the ground with the toes of the affected leg; nutrition and sensation of the limb natural. The gastrocnemii, the extensor longus, and flexor longus digitorum pedis, &c. have not as yet resumed their natural action, so that he cannot well move his foot.

The hemorrhage on the third day from the operation, (which indeed had begun a few hours after it,) was produced by the looseness of the upper ligature, which I can only account for by my having slightly pulled up the two ends of it after the first twist had been tied, to make the second,—which might have slackened the first. In fact, the oozing of the blood appeared soon after the patient recovered from fainting; but, on account of the tightness of the dressings, and of the slowness of the oozing, the blood coagulated, and opposed a temporary obstacle to further hemorrhage. When the dressings, and consequently the pressure, were removed, the ligature was cast off altogether, and hemorrhage ensued. Similar occurrences are not unfrequent; they have happened to Sir A. Cooper, to Mr. Cline, and to others.*

The opening and emptying of the aneurismal sac was productive of a great benefit in promoting the absorption of the fluids producing œdema, and in checking the sphacelus, which, after all, was produced more from pressure than from impeded circulation.

The ligatures seem to have undergone no change whatever, notwithstanding having remained for nearly three months among living parts. Very likely the third ligature will be discharged at some future period.

Had it not been for the upper loose ligature, I am confident that the operation wound would have healed by the first intention, and the cure shortened by many weeks. This case proves the safety of operating for aneurism in the same way (with regard to ligatures) as for amputations, viz. cutting both ends of them close to the knot; while, at the same time, it points out very strongly the necessity of great care in securing the ligatures, especially the upper one. It seems also to show that the silk is not absorbed, but generally finds its way out, sooner or later, through the cicatrix.

*Corfu; 6th February,* 1826.

---

* Vide Cooper's *Surgical Dictionary,* article Aneurism.

